# Overview of Venous Thromboembolism and Emerging Therapeutic Technologies Based on Nanocarriers-Mediated Drug Delivery Systems

**DOI:** 10.3390/molecules29204883

**Published:** 2024-10-15

**Authors:** Masoud Salavati, Arman Arabshomali, Sasan Nouranian, Zia Shariat-Madar

**Affiliations:** 1 Department of Chemical Engineering, University of Mississippi, Oxford, MS 38677, USA; msalavat@go.olemiss.edu (M.S.); sasan@olemiss.edu (S.N.); 2 Pharmacy Administration, School of Pharmacy, University of Mississippi, Oxford, MS 38677, USA; marabsho@go.olemiss.edu; 3 Division of Pharmacology, School of Pharmacy, University of Mississippi, Oxford, MS 38677, USA

**Keywords:** thrombotic disorders, targeted-antithrombotic approaches, nanothrombolysis, clot-penetrating drug, aging, fibrinolysis

## Abstract

Venous thromboembolism (VTE) is a serious health condition and represents an important cause of morbidity and, in some cases, mortality due to the lack of effective treatment options. According to the Centers for Disease Control and Prevention, 3 out of 10 people with VTE will have recurrence of a clotting event within ten years, presenting a significant unmet medical need. For some VTE patients, symptoms can last longer and have a higher than average risk of serious complications; in contrast, others may experience complications arising from insufficient therapies. People with VTE are initially treated with anticoagulants to prevent conditions such as stroke and to reduce the recurrence of VTE. However, thrombolytic therapy is used for people with pulmonary embolism (PE) experiencing low blood pressure or in severe cases of DVT. New drugs are under development, with the aim to ensure they are safe and effective, and may provide an additional option for the treatment of VTE. In this review, we summarize all ongoing trials evaluating anticoagulant interventions in VTE listed in clinicaltrials.gov, clarifying their underlying mechanisms and evaluating whether they prevent the progression of DVT to PE and recurrence of thrombosis. Moreover, this review summarizes the available evidence that supports the use of antiplatelet therapy for VTE. Since thrombolytic agents would cause off-target effects, targeted drug delivery platforms are used to develop various therapeutics for thrombotic diseases. We discuss the recent advances achieved with thrombus-targeting nanocarriers as well as the major challenges associated with the use of nanoparticle-based therapeutics.

## 1. Introduction

VTE is one of the most common non-malignant blood disorders in the United States (U.S.) [1] and in most countries [2], representing a serious problem for our national and global health. Although VTE has had much progress over the past decade, one in twelve people will be expected to experience VTE in their lifetime [3]. It is estimated that 20% of patients with VTE are at increased risk of dying, particularly within the first year after diagnosis [3,4]. The pathophysiology of VTE includes stasis, endothelial injury, and hypercoagulability, which were described by Rudolf Virchow [5]. VTE is associated with a variety of interrelated biological processes and functional alterations [6], but remains associated with derangements in hemostasis. VTE covers two interrelated clinical events: (1) pulmonary embolism (PE), a blockage in the lung, and (2) deep vein thrombosis (DVT), a blood clot that develops within a vein [7]. Below are brief descriptions of these two blood clot conditions, which can be fatal if left untreated.

DVT is characterized by the formation of blood clots (thrombi) in the deep veins [8,9,10], which can be categorized as acute, subacute, and chronic. During these periods, acute thrombi are considered to be prone to detaching from the vein, leading to a higher risk of PE. DVT is considered a multifactorial disease, indicating that many genetics and provoking factors such as immobilization and surgery affect the initiation and continuance of the disease [11]. Recent evidence shows that plasminogen activator inhibitor-1 (PAI-1) polymorphism -675 ID, 4G/5G, factor V Leiden mutation, and prothrombin G20210A single nucleotide polymorphism (SNP) have been correlated with elevated DVT levels [12]. The von Willebrand factor (vWF) has a vital role in the pathological process of thrombus formation. vWF, a blood-clotting protein, is involved in platelet aggregation and adhesion, and it also serves as a carrier for factor VIII, a risk factor for VTE, from proteolytic degradation [13]. Unsurprisingly, SNPs in seven genes associated with vWF were found to be independently associated with incident venous thrombosis [14]. While DVT is a severe clinical entity, it resolves spontaneously without complications. Given that DVT is relatively common [15] and associated with significant morbidity and mortality [16], significant progress has been made in order to develop new therapies and clinical evaluations to control DVT and reduce immediate complications.

PE occurs when a segment of a DVT clot breaks off and blocks a pulmonary artery in the lung. This reduces blood flow in the artery, which may cause severe shortness of breath and chest pain. The risk factors for PE are similar to those of DVT. There are numerous risk factors for PE [17], including family history of clots, old age, cancer, use of estrogen-containing oral contraceptives, pregnancy, and surgery. A study indicates that two out of three individuals with PE die within two hours after presentation [17]. This indicates that some PE patients present a type lacking an obvious identifiable cause. Efforts were made to detect genetic variants affecting PE levels through genome-wide association studies [18]. This study showed that there were strong associations between PE and the polymorphisms methylenetetrahydrofolate reductase (MTHFR) C677T, PAI-1 4G/5G, and ITGB3 (Glanzmann thrombasthenia), suggesting PE is a different phenotype in VTE genetic studies [18]. Another study has found associations between PE and the polymorphisms factor V, factor XIII, beta fibrinogen, and TFPI [19]. Although these studies show relationships between several genetic variants and PE, the exact mechanisms remain unclear. Interestingly, the aforementioned genetic risk factors for PE patients are not identical to those of DVT patients.

VTE coexists with numerous chronic diseases, which share risk factors such as age, obesity, metabolic syndrome, and cardiovascular diseases. Diabetes [20], cancer [21,22,23], and acquired immunodeficiency syndrome (AIDS) are the known risk factors for VTE [24]. VTE is considered to be a common medical disorder complicating pregnancy [25,26]. Evidence clearly indicates that drug, sex [27], and hospitalization [28] interact with the risk of developing VTE in patients with chronic disorders. Patients with chronic kidney disease exhibit bleeding and are prone to thrombosis. While the risk of developing VTE is highest after major surgery or injury, both immunotherapy [29] and anti-angiogenic drugs [30,31,32] are shown to cause an increased risk of VTE. The abovementioned studies indicate that VTE can happen to anyone.

PubMed/MEDLINE, Scopus, Cochrane Library, and Clinicaltrials.gov were searched using search terms to retrieve relevant, topic-specific results from inception to 25 August 2024. Cohort studies, case-controlled case reports, and randomized controlled trials evaluating the effect of any anticoagulants, antiplatelet agents, and fibrinolytic agents on DTV, thrombus, and emboli were included. There were two key elements in our search. First, we aimed to identify articles that describe the recent advances in coagulation cascade, fibrinolysis, and in platelet function; and second, to identify articles that are appropriate to use for clinical decision-making at the point of care. Studies that reported alteration in prothrombin time (PT), activated partial prothrombin time (aPTT), thrombin time (TT), D-dimer and CRP levels, urinary thromboxane A2, thrombus size, and blood clot recurrence after a DVT for hospitalized patients and postdischarge extended-duration thromboprophylaxis for prevention of VTE in patients.

The goal of antithrombotic therapy is to reduce cardiovascular and cerebrovascular morbidity, prevent the likelihood of further VTE recurrence, help improve blood flow in the blood vessels, and prevent life-threatening events such as PE and other forms of thromboembolic events. We outline the recent advances in managing VTE and medications used to help pharmacological management, such as preventing fibrin formation, slowing down blood clots, dissolving existing blood clots, and treating thrombosis in VTE patients. We focus on the use of anticoagulants, antiplatelet agents, thrombolytic agents, and anti-fibrinolytic drugs in clinical trials. We specifically aim to highlight the potential of targeted drug delivery systems in enhancing the targeting and efficacy of new and existing anticoagulant therapy and ultimately improving patient outcomes.

## 2. Blood Coagulation System

**Figure 1:**
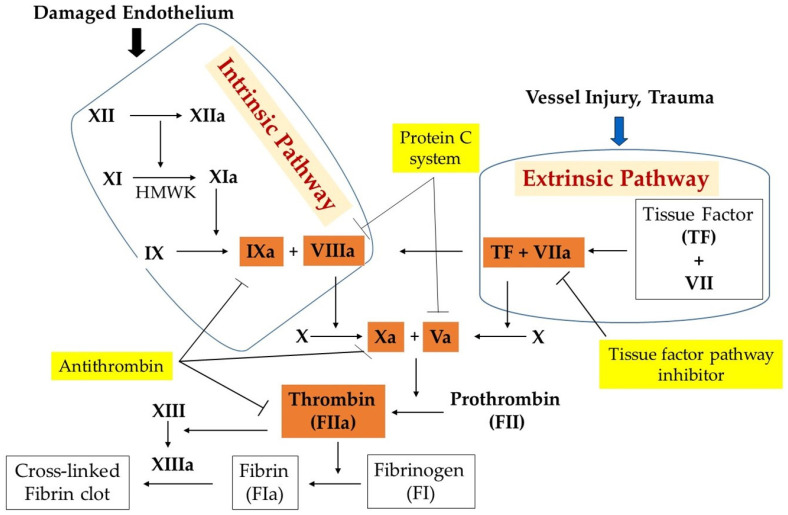
Coagulation system. It responds to vascular injury to induce blood clot formation and prevent excessive bleeding.

Blood coagulation factors constitute a key class of proteases and cofactors that serve collectively, as illustrated in Figure 1. Coagulation proteases are synthesized as inactive precursors (zymogens) in the liver and are then secreted into the blood. Upon vascular injury, the zymogens are converted to the active form by the selective enzymatic cleavage of the peptide bond(s). The blood coagulation proteases are different from each other in terms of zymogen substrate specificity, active site, and catalytic mechanism. The sequence of consecutive activation of the coagulation reaction is governed by the specificity of each protease, ions, and cofactors, whereas the degree of amplification is linked to the activation of an effector molecule downstream in the pathway. The blood coagulation pathway is divided into two phases: Phase 1 (extrinsic pathway) and Phase 2 (intrinsic pathway) (Figure 1). The extrinsic pathway includes the interaction between the transmembrane receptor tissue factor (TF) and plasma factor VII/VIIa (FVII/FVIIa) [33]. The binding of TF to FVII results in the conversion of zymogen FVII to activated factor VII (FVIIa). Evidence indicates that TF enhances FVIIa catalytic activity [34]. The TF:FVII complex is also activated by several proteases including thrombin, an effector in coagulation. The formed TF:FVIIa complex consequently activates factor IX (FIX) to activated factor IX (FIXa) and factor X (FX) to activated factor X (FXa) on the platelet surface in the presence of calcium. The intrinsic pathway includes the interaction between plasma factor XI (FXI), factor IX (FIX), and factor VIII (FVIII). Although different proteases can activate zymogen FXI to activated FXI (FXIa), thrombin and activated factor XII (FXIIa) are highly effective in mediating FXI activation. FXIa activates FIX to activated FIX (FIXa) and consequently activates factor X in the presence of activated factor VIII (FVIIIa) and calcium on the surface of a platelet. The effectors of both extrinsic and intrinsic pathways activate factor X (FX), a converging target, which integrates their function. Thus, factor Xase has emerged as a key mediator of coagulation. FXa activates factor II (prothrombin) into activated factor II (thrombin) in the presence of cofactor Va and calcium ions, the components of the common pathway. Thrombin plays a crucial role in converting factor I (fibrinogen) into fibrin, an insoluble fiber. Thrombin activates factor XIII into activated factor XIII (XIIIa), a calcium-dependent transglutaminase. FXIIIa cross-links fibrin monomers to stabilize the platelet plug and form a thrombus, a blood clot.

The central dogma states that blood clot formation normally occurs when the blood coagulation proteins are activated in a specific sequence to convert fibrinogen to fibrin. As a consequence, it has been generally assumed that these proteins fulfil not only catalytic but also regulatory functions in stopping bleeding at the site of vessel injury. However, recent startling findings state that hemostasis is highly regulated by cellular function. Here we briefly summarize the newer model. The cell-based model of coagulation, proposed by Hoffman and Monroe [35] in 2001, revolutionized our understanding of hemostasis by emphasizing the crucial role of cellular components in the coagulation process. This model delineates coagulation into three overlapping phases: initiation, amplification, and propagation, each occurring on specific cell surfaces [36]. The initiation phase begins on tissue factor-bearing cells, generating small amounts of thrombin. During amplification, platelets and cofactors are activated, preparing for large-scale thrombin generation. Finally, the propagation phase occurs on activated platelet surfaces, resulting in a burst of thrombin production. This model provides a more accurate representation of in vivo hemostasis compared to the traditional cascade model, highlighting the importance of cellular control in coagulation [37].

The cell-based model of coagulation has had significant implications for the development of antithrombotic therapies. By emphasizing the role of specific cell surfaces and receptors in controlling coagulation, this model has enabled the identification of novel targets for anticoagulant drugs [38]. For instance, the recognition of factor Xa as a crucial component in both the initiation and propagation phases led to the development of direct oral factor Xa inhibitors, such as rivaroxaban and apixaban [38]. These drugs offer more predictable pharmacokinetics and pharmacodynamics compared to traditional anticoagulants like warfarin, with fewer food–drug interactions and no need for routine monitoring [38]. Additionally, the cell-based model has improved our understanding of the pathophysiology of coagulation disorders, allowing for more targeted therapeutic approaches [36]. For example, insights into the mechanism of action of recombinant factor VIIa in hemophilia patients with inhibitors were derived from this model, leading to its successful clinical application [36]. Furthermore, the enhanced understanding of cell-free histones (CFHs) and other damage-associated molecular patterns (DAMPs) in the cell-based model of coagulation offers promising avenues for innovative therapies in areas where immunothrombotic complications are prevalent and current treatments are inadequate [37]. Readers are referred to a great review article by Yong et al. [37] for a more comprehensive understanding of the roles of CFHs and DAMPs in coagulation and thrombosis.

## 3. Fibrinolysis

**Figure 2:**
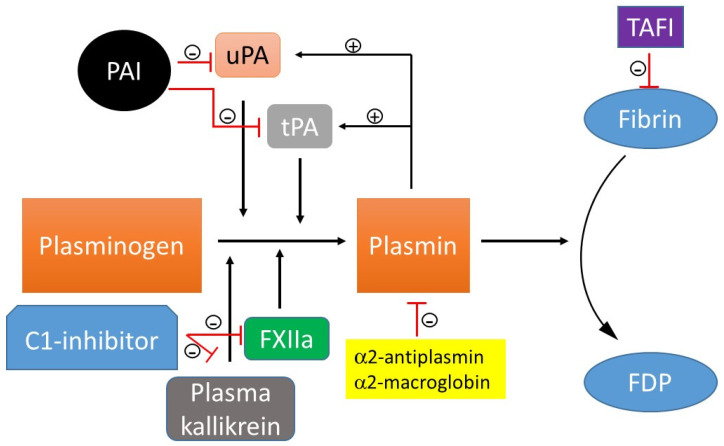
Course of fibrinolytic activity and inhibition. Fibrinolysis is the process of dissolving blood clots. Tissue- or urokinase-type plasminogen activator (tPA, uPA), kallikrein, and activated factor XII (FXIIa) can cause the transformation of plasminogen into plasmin. Formed plasmin degrades the deposited fibrin into fibrin degradation products (FDP). Plasminogen activator inhibitor -1 (PAI-1) and plasminogen activator inhibitor-2 inhibit both tPA and uPA, whereas C1- esterase inhibitor (C1-inhibitor) modulates both plasma kallikrein and FXIIa. α2-Antiplasmin is a direct inhibitor of plasmin; α2-macroglobulin is a minor plasmin inhibitor. Activated thrombin-activated fibrinolysis inhibitor (TAFI) protects the fibrin clot against lysis.

The opposite to thrombosis is fibrinolysis. Plasmin is the main protease that drives the progression of fibrinolysis. Plasmin is a molecular switch that, when activated from plasminogen by tissue plasminogen activator (tPA), urokinase-type plasminogen activator (uPA), kallikrein, XIIa, can cause the conversion of fibrin into fibrin degradation product (FDP) (Figure 2). FXII, tPA, and uPA are spatially synthesized by different cell types. FXII is synthesized by the liver. While tPA is synthesized by endothelial cells, uPA is synthesized by monocytes, macrophages, and urinary epithelial cells. Fibrinolysis is, in turn, temporally coordinated with these proteolytic processes. Plasmin activates both tPA and uPA, creating a positive feedback loop that triggers a brief impetus to produce a sustained conversion of inactive plasminogen to active plasmin. This protective feedback loop prevents unnecessary accumulation of intravascular fibrin and ensures the blood has sufficient plasmin needed to remove the thrombus. Excellent reviews on the history of the discovery of fibrinolysis, current understanding of the fibrinolytic system, and the fibrinolytic system as a treatment target can also be found elsewhere [39,40].

Specific inhibitors can bind to circulating plasmin and plasminogen activators (uPA, tPA), preventing them from engaging in excessive unregulated activities and thus maintaining the integrity of the thrombus (Figure 2). Plasminogen activator inhibitor (PAI), a serpin inhibitor, inhibits both tPA and uPA [41]. Plasmin is neutralized by α2-antiplasmin, a plasma serpin; α2-antiplasmin is incapable of inhibiting plasmin when it is bound to fibrin. Plasmin is modulated by α2-macroglobin, a nonspecific protease inactivator. C1-esterase inhibitor, a physiological inhibitor of plasma kallikrein and FXIIa, also attenuates the nonspecific plasminogen activation [42,43]. Moreover, thrombin-activated fibrinolysis inhibitor (TAFI), a non-serpin inhibitor, removes C-terminal lysine and arginine residues on fibrin. This decreases the number of available plasminogen binding sites, leading to a reduction in plasmin formation and clot stabilization. The reader is referred to the following articles for more detailed information on TAFI [44,45]. Fibrinolysis is tightly regulated by the complex interplay of activating and inhibitory pathways.

## 4. The Physiology of Thrombosis

The procoagulant/anticoagulant balance is maintained through the interaction between platelets and vascular endothelium, and with a predominance of the active form of anticoagulant forces. Coagulation activation is tightly regulated by several endogenous anticoagulants, which provide a counter mechanism to clot formation. Tissue factor pathway inhibitors (TFPI) and thrombomodulin are endothelial-associated anticoagulant proteins, while protein C, protein S, and antithrombin are circulating anticoagulant proteins. The TFPI pathway inhibits early phases of the procoagulant response [46], whereas activated protein C in concert with its cofactor, protein S, degrade factors VIIIa and Va [47], the two proteins that play an important role in the final step of the coagulation pathway. The partial deficiencies of the circulating anticoagulant proteins are causes of VTE [48], indicating that the anticoagulation system is responsible for regulating hemostasis. The reader is referred to the following reviews for more detailed information [49,50,51,52] (Figure 1). Procoagulant/anticoagulant imbalance leads to the pathogenesis of stroke, ischemia, myocardial infarction, and particularly venous thromboembolism. Increasing levels of the active form of procoagulants due to reduced anticoagulants and perturbed vascular endothelium cause unchecked activation of coagulation. It has emerged that endothelial dysfunction and hemostatic derangement cause several pathophysiological disturbances of primary and secondary hemostasis, fibrinolysis, and subsequent tissue repair.

**Figure 3:**
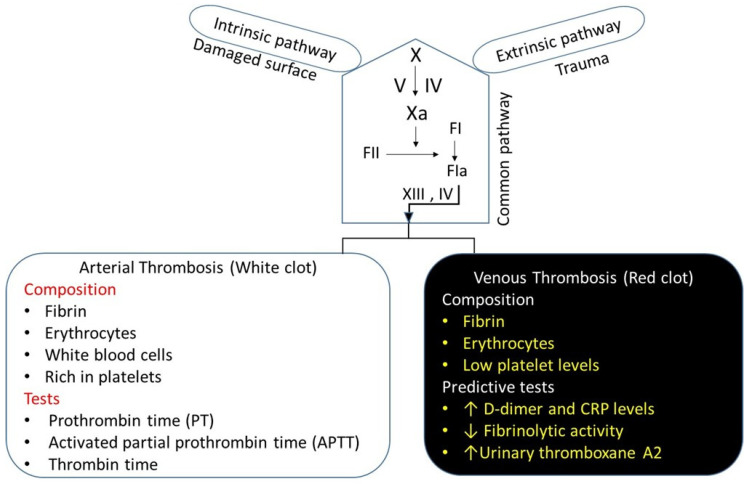
The coagulation pathways and thrombosis. The bleeding time is a screening test for the arterial phase of hemostasis. Three in vitro tests are used to measure the time elapsed from activation of the intrinsic, extrinsic, and common pathways of the coagulation system. Commonly used tests for arterial thrombosis are as follows—prothrombin time (PT), activated partial prothrombin time (APTT), and thrombin time (TT). Similarly, three in vitro predictive tests are used for the venous phase of hemostasis. Tests used to diagnose or rule out DVT include: D-dimer blood test and CRP levels, fibrinolytic testing, and urinary thromboxane test.

There are two broad classifications of thrombosis: arterial thrombosis and venous thrombosis, which is a major focus of this review (Figure 3). Both arterial thrombosis and venous thrombosis are similar, but there is a subtle difference between the two. Arterial thrombosis is a platelet-predominant phenomenon. Histopathologic features that have been considered specific for arterial thrombosis include fibrin, leukocytes, and an abundance of platelets, providing a white appearance [53]. Patients with arterial thrombi are at high risk of acute stroke, myocardial infarction, or peripheral arterial disease. Venous thrombosis is generally understood as a disorder in plasma coagulation, and is associated with red blood cells and fibrin-rich, presenting a red appearance, i.e., as a “red clot” [54] (Figure 3). Venous thrombi occur in areas of reduced blood flow and stasis, leading to the accumulation of procoagulants and reduced levels of the local anticoagulant pathway [55]. The severe compression of the iliac vein in pregnant women [56] patients, May–Thurner syndrome (an iliac vein compression syndrome) [57], or the combination of the two, creates venous stasis that can contribute to the increased risk of VTE. On histopathology, venous clots are composed of fibrin, leukocytes, and red blood cells, and low levels of platelets compared to arterial thrombi. According to the dogma, platelets are not involved in the pathogenesis of venous thrombosis. However, recent evidence suggests that platelets play an important role in VTE pathophysiology [58], and may be involved in the initiation of DVT [59]. This suggests a need to consider the antiplatelet agents as antithrombotic therapy for the management of DVT and VTE.

The occurrence of a thrombus is determined by a physical exam, imaging tests, and the procoagulant/anticoagulant balance. Various provoking factors that cause thrombus formation disrupt the procoagulant/anticoagulant balance. These provoking factors include: (1) medical devices (vascular grafts, stents, catheters, and heart valves); (2) chronic diseases (chronic kidney disease, heart disease, uncontrolled hypertension, chronic inflammation, diabetes, cancer, old age); (3) cell perturbation (activated endothelial cells, activation of blood platelets); (4) lifestyle (being overweight or obese, smoking); and (5) medications (oral contraceptives or hormone replacement therapy).

## 5. Pharmacotherapy of VTE: An Update

**Figure 4:**
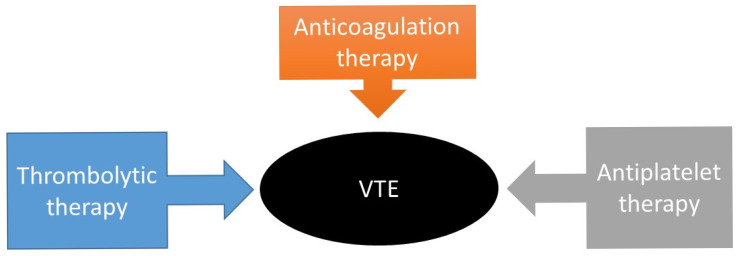
The conventional treatments for VTE.

This section provides an overview of the drugs used for the treatment of VTE. Anticoagulation therapy, thrombolytic therapy, and antiplatelet therapy are considered the standard of care for VTE treatment (Figure 4). The American Society of Hematology and the American College of Chest Physicians (ACCP) strongly recommend anticoagulation therapy. Anticoagulants as first-line medications can be used to prevent, treat, and reduce the recurrence of VTE, and help to prevent stroke in persons with atrial fibrillation [60]. Anticoagulants are a class of medications that block key serine proteases.

The conventional drugs used in the treatment of DVT are blood thinners, also called anticoagulants, that control thrombus formation. Typically, anticoagulants [61] directly or indirectly inhibit the formation or activity of thrombin involved in maintaining the clot-forming process [61], although they may also contribute to other hemostatic benefits. These include (1) heparin, an indirect thrombin inhibitor that stops blood from forming blood clots or prevents the formation of a bigger blood clot; (2) warfarin, a vitamin K antagonist used as an oral anticoagulant that is prescribed following the initial treatment to prevent another blood clot; (3) low molecular weight heparin (Enoxaparin, Apixaban, Betrixaban, Dabigatran, Edoxaban, and Rivaroxaban); and (4) Fondaprinux, a synthetic heparin pentasaccharide, used to prevent VTE and treat DVT and improve survival following myocardial infarction. Indiscriminate distribution of most anticoagulants by systemic administration and lack of specificity to the DVT may contribute to an increased risk of bleeding. In addition, since most of the anticoagulants are removed via the renal system, thereby these drugs pose an increased risk of chronic kidney disease.

A combination of heparin and oral anticoagulants is commonly used for the prevention and treatment of patients with acute DVT [62]. Although highly effective, they are also associated with significant bleeding risks [63]. Remarkably, new antidotes that inactivate heparins and direct oral anticoagulants have been recently tried with success [64,65,66,67]. The management of DVT still remains a major challenge. In other words, none of the currently available risk factor tools [68] is effective enough in predicting bleeding including intracranial hemorrhage. American Society of Hematology recommendations include the following: (a) use of thrombolytic therapy for patients with PE and hemodynamic compromise to rapidly dissolve the embolic burden; (b) the target international normalized ratio (INR) should be between 2.0 to 3.0 for the majority of patients with PE [69]. A low INR range is recommended for patients with VTE receiving a vitamin K antagonist for secondary prevention; and (c) use of indefinite anticoagulation for persons with recurrent unprovoked VTE or associated with chronic factors [7]. Antiplatelet agents appear to reduce recurrent VTE [70,71]. In a meta-analysis of randomized studies by the Antiplatelet Trialists’ collaboration in 1994, antiplatelet therapy was shown to reduce the risk of VTE [72]. Some patients will require combinational therapy to produce better outcomes. Many of the above medicines used to treat VTE are associated with unwanted bleeding as a side effect. Table 1 outlines the many new antithrombotic drugs that target various steps in the hemostatic system. The direct, small-molecule inhibitors of coagulation proteins (e.g., factor Xa, thrombin) [73] presented in this table have similar safety profiles compared with antithrombin-dependent heparins. Table 2 summarizes the recommended drug classes according to compelling indications.

## 6. Drugs in Development for VTE

We conducted a comprehensive search on the clinicaltrials.gov website to identify candidate drugs for VTE and related complications that had progressed through phases 1–3 of clinical trials, with their results posted within the past five years. Numerous VTE studies seek to develop new therapeutics, targeted therapeutic drugs, and technologies to improve efficacy and safety compared with traditional VTE drugs. In this section, we present important drug candidates for each target in clinical trials. These agents target coagulation factors (FXI, thrombin), modulate the endogenous inhibitors (protein C) of the blood coagulation factors, activated thrombin activatable fibrinolysis inhibitor (a regulator of fibrinolysis, or the P-selectin/PSGL-1 pathway, which has a pivotal role in both thromboinflammation and immunothrombosis (Table 3). We briefly discuss the significance of FXI and P-selectin glycoprotein ligand 1 (PSGL-1) to demonstrate how they affect thrombus formation. We also provide a summary of recent targeted therapeutic delivery methods of different nanomaterials and technologies that have shown significant promise for treating VTE and DVT.

### 6.1. FXI as a Novel Drug Target

**Figure 5:**
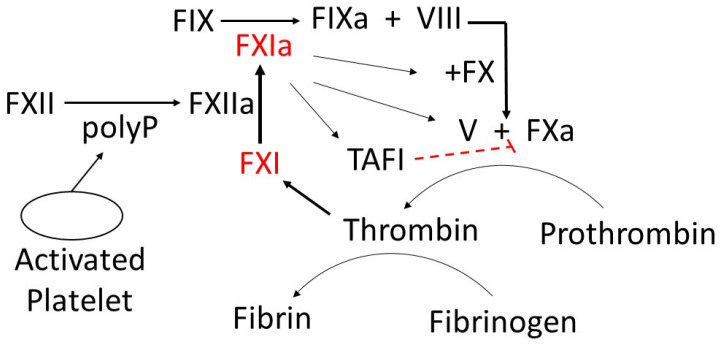
Activation and prothrombotic activity of active factor XI (FXIa).

FXIa inhibitors significantly lessen subsequent or recurrent embolization [97]. Because FXI deficiency (Hemophilia C, Rosenthal Syndrome) does not manifest clinically significant bleeding disorders, one might hypothesize that FXI would be a relatively poor activator of FX. Nonetheless, this is not the case. FXI, when cleaved by FXIIa (Hageman factor), forms activated factor XI (FXIa), which then activates factor IX (FX)—mediated active FX (FXa) generation (Figure 5). FXa converts prothrombin to thrombin, a protease that converts fibrinogen to fibrin, which is responsible for trapping platelets and holding a clot in place (Figure 5). Both FXI and its substrate, factor IX (FIX) protease, are increased in VTE [98]. FXIa/FIX complex coupled with its abundance in the blood, indicates that FXIa plays a significant role in promoting thrombus formation and that modest increases in this protease can profoundly enhance clot formation. The procoagulant property of FXIa has been studied intensively in the venous system of experimental models [99]. Patients with FXI deficiency rarely manifest hemarthroses and muscle hematomas.

When the endothelium of a blood vessel is damaged, exposing the subendothelium to blood, both platelets and FXII are activated, promoting blood coagulation. Exposure of FXII to collagen leads to activation of FXII-mediated FXIa generation. Platelet-derived polyphosphate (polyP) can potentiate thrombin-feedback pathways through FXIa-mediated FIXa generation (Figure 5). Recent evidence indicates that FXIa contributes to clot formation by directly activating FX and FV [100]. The activation of FXI is also increased in the presence of thrombin [8], suggesting that thrombin circumvents the need for FXIIa to activate FXI. A positive feedback loop connected by the FXIa-mediated coagulation pathway promotes more fibrin formation (Figure 5). This positive feedback loop, which enhances thrombin generation, is relevant to the role of active FXI in promoting clot formation and maintaining clot stabilization. Active FXI also contributes to thrombosis through indirect activation of thrombin activatable fibrinolysis inhibitor (TAFI), which can contract fibrinolysis and pose a threat to the patient (Figure 5) [101]. An increase in FXIa-mediated activation leading to higher concentrations of active FIX, FX, thrombin [102], and TAFI can disrupt the normal equilibrium, resulting in an increased tendency to form venous thrombi. Thus, inhibition of FXIa is physiologically relevant in contexts where its concentration is elevated, such as at the site of clot formation, where thrombin is formed. Since a complete deficiency of FX in mice results in a lethal phenotype [103], inhibitors of FXIa can reduce the overconsumption of both FIX-dependent and FIX-independent FXa generation.

Considerable progress has been made in the recent decade toward the successful development of an oral FXIa inhibitor, which could safely provide increased benefit and be used in combination with other antithrombotics for various thrombotic indications. FXIa has been successfully targeted with monoclonal antibodies, aptamers, antisense oligonucleotide inhibitors (ASOIs), natural peptides, and small synthetic molecules [104,105]. However, few of them have reached clinical trials or market. Given the existence of several current review articles [97,105,106,107,108,109] that comprehensively cover the pharmacology and clinical efficacy of these inhibitors, we refer readers to these sources for detailed information. Phase 2 studies of different classes of FXI inhibitors are promising [110]. A recent clinical study shows that ASOIs against FXI prevent venous thrombosis in elective knee surgery. The inhibitors of FXI or FXIa are found to be effective in phase 2 studies at preventing VTE [111].

### 6.2. P-Selectin/PSGL-1 Pathway

Platelets have numerous adhesion proteins that are involved in platelet–platelet, platelet–endothelial, platelet–monocyte, platelet–-leukocyte, and platelet–extracellular matrix (ECM) interactions. Upon activation during the course of both inflammatory and thrombogenic responses, P-selectin is released from α-granules of platelets, and from Weibel–Palade bodies of endothelial cells. P-selectin is translocated to the cell surface. P-selectin has a high affinity for PSGL-1, which is expressed on the surfaces of all leukocytes, facilitating the initial “capturing and rolling” step in the leukocyte–endothelial cell adhesion cascade [112] and adhesion in an early phase of platelet activation to limit bleeding by promoting platelet aggregation and thrombosis. Activated platelets are also capable of forming circulating aggregates with leukocytes [113] and monocytes [114] to promote systemic inflammatory responses. The inhibitors of the P-selectin/PSGL-1 pathway have the potential to reduce thrombosis and inflammation (Table 3).

Excluding factor XI inhibitors, our search yielded 33 studies primarily involving modifications to existing drugs, such as variations in indications, dosage forms, dose schedules, or combinations thereof. Among these studies, we identified four new lead compounds. The summary of these potential new treatments is shown in (Table 3).

**Table 3:** New compounds for VTE identified from recent clinical trials.

Conditions	Compounds	Mechanism of Action	Phase	Sponsor	Ref.
Pulmonary embolism; thrombotic disease	DS-1040b	Inhibits the activated form of thrombin-activatable fibrinolysis inhibitor (TAFIa).	Phase 1|Phase 2	Daiichi Sankyo	[115,116]
VTE prophylaxis with anticoagulation after total knee replacement surgery	JNJ-64179375	Specific exosite 1 thrombin inhibitor	Phase 2	Janssen Research & Development, LLC Tetherex	[117,118]
VTE	SelK2	Targets PSGL-1 and blocks its interactions	Phase 2	Pharmaceuticals Corporation	[119]
Thromboembolism of vein VTE in colorectal cancer, pancreatic cancer, non-small cell lung cancer	Isoquercetin	Decreases D-dimer, P-selectin, and platelet-dependent fibrin generation	Phase 2|Phase 3	Quercegen Pharmaceutical; National Heart, Lung, and Blood Institute (NHLBI)	[120]

### 6.3. New Modes of Delivery for Anti-Thrombotic Drugs

While many thrombi spontaneously lyse, the fate of a thrombus depends on the persistence of increased activation of the extrinsic pathway, intrinsic pathway, or reduced levels of endogenous coagulation inhibitors, such as protein C or antithrombin. Reduced levels of coagulant proteins can lead to hemorrhage [121,122]. Targeting the blood coagulation proteases therapeutically has not been an easy task due to the connectivity among them, their trypsin-like specificity, protein–protein interaction, protein–serpin interaction, protein–lipid interaction, and ions.

Although the standard anticoagulant treatment for DVT has shown remarkable progress in recent years, thrombolytic therapy has been found to be more effective in dissolving dangerous intravascular clots completely compared to anticoagulant treatment. A major advantage of thrombolytic therapy is its capability to prevent ischemic damage by improving blood flow [123]. Like anticoagulant drugs, thrombolytic drugs have inadequate efficacy and are associated with an increased risk of hemorrhage, implying that there is room for improvement. Polymeric nanocarriers are being developed to achieve the protection and targeted delivery of thrombotic drugs [124]. Because various functional groups can be added to nanocarrier systems that are sensitive to the specific physiological environment in the circulatory system [125], they can be used in thrombus-targeted treatments. Nanocarrier-based delivery systems not only protect drugs from rapid degradation or excretion, but they are also capable of enhancing bioavailability and drug efficacy with respect to free drug formulations. Furthermore, nanocarrier systems exhibit robust successes in targeted delivery of antithrombotic drugs to the desired tissues and cells and enhanced intracellular delivery. In this section, we review FXI and the recent nanocarrier-based delivery systems that can offer solutions to common problems faced by several anticoagulant and thrombotic treatments. These include the following: (1) polymers; (2) dendrimers (3) PEGylation; (4) liposomes; (5) echogenic liposomes; (6) polymeric nanoparticles; (7) mechanically activated nanotherapeutics; (8) fucoidan-functionalized nanocomplex; (9) platelet-based drug delivery system; and (10) combinational therapy.

#### 6.3.1. Polymers

Although anticoagulant therapy remains the first line of treatment for VTE, the efficiency of treatment via optimization of more effectively delivering a therapeutic agent to a target site remains a major goal of current scientific research. Therapeutic antibodies, proteins, and nucleic acids are highly susceptible to oxidation, aggregation, degradation, and elimination. Over the past decade, the therapeutic approach of biological therapy (macromolecules or biologics) has gained momentum due to its potentially superior effects compared with small molecules. They exhibit much higher specificity than small molecule drugs: they tend to be more efficient, safer, and cause fewer unwanted side effects. Recent advances in nanomedicine (nanostructured polymers) offer new tools for delivering and maintaining therapeutic concentrations of drugs and releasing them (passively or actively) at the target site. The U.S. Food and Drug Administration (FDA) basically describes nanomedicines as products in the nanoscale range (1−100 nm) [126]. While nanomedicine with 10 nm products is possible in several compartments, including blood, spleen, kidney, testis, thymus, heart, lung, and brain, fewer larger ones are detected in blood, spleen, and liver [127]. Effective and long-acting nanomedicines have shown enormous potential as therapeutics for the treatment and management of various human diseases, including pain, advanced prostate cancer, tumor-targeted delivery, Alzheimer’s disease, and Parkinson’s disease. Nanomedicine encompasses a suitable biodegradable polymer, a carrier, and an active drug or a prodrug. The drugs are generally conjugated or encapsulated by polymeric nanocarriers. A drug–polymeric nanocarrier complex is known as polymeric nanomedicine Table 4). Nanobiopolymers, products biosynthesized by living organisms, are emerging as a promising class of materials that play a vital role within the field of medicine, including controlled cancer drug delivery and gene delivery. Classification of nanomedicine platforms is based on their nanocarriers such as organic (polymer-based and lipid-based), inorganic, crystalline, or amorphous particles [128]. Organic nanomaterials (lipid-based systems) are characterized as one of the most promising innovative polymeric nanocarriers, which are now FDA-approved [129] due to their respective therapeutic utilities [130]. Organic nanomaterials exhibit a variety of different architectures, including micelles, liposomes, dendrimers, polymersomes, hydrogels, and metal–organic compounds. Recently, organic nanomaterials have been used for the development of polymeric nanobiopolymers to attain a thrombolytic drug-loading system (Table 4). Although considerable progress has been made in the development of polymeric nanocarriers that encapsulate and deliver a controlled release of the drug to the thrombus site, the clinical translation of polymeric nanocarriers is limited.

#### 6.3.2. PEGylation

PEGylation is a process where polyethylene glycol (PEG) chains are attached to molecules or proteins, and it has been used in the development of numerous therapies in the clinic. PEGylated drugs are shown to have increased circulation time [165], decreased immunogenicity [166], improved stability [167], solubility [168], bioavailability [168], and targeting and delivery [169]. However, the size of the PEG group itself can have unfavorable impacts on either the activity or pharmacokinetic behavior of the drug [170]. Other potential drawbacks of the clinical translation of PEGylated therapeutic proteins are hindered by manufacturing challenges, intracellular uptake of PEG, size enlargement, release of PEG antibodies, and reduced activity of the drug [171].

Recently, PEGylation with various biophysiochemical properties has been designed to explore drug-targeting delivery carriers of different anti-thrombotic drugs for the treatment of DVT. PEGylation for thrombolysis is used for a wide range of drugs such as tissue plasminogen activator (tPA) [172,173], staphylokinase (SAK) [136], urokinase (UK) [174], and streptokinase (SK) (Table 4) [175]. Qi et al. [136] utilized PEG maleimide to conjugate multiple staphylokinase (SAK) molecules, aiming to enhance their bioactivity and extend their half-life. However, the bioactive domain of SAK was unable to interact with its receptor effectively due to the steric shielding effect of PEG, which resulted in reduced bioactivity of SAK. Despite this, the conjugation improved the thermal stability of SAK molecules and did not cause detectable harm to the liver, kidney, or heart in mice. Zamanlu et al. [173] utilized PEGylation to create polymer–protein nanoparticles using poly(lactic-co-glycolic acid) (PLGA) and tPA. The study concluded that this approach may improve the biological half-life of tPA, potentially addressing clinical challenges and allowing for controlled and predictable drug release kinetics [136]. Developing improved therapies for VTE and other thrombotic conditions presents significant challenges. To enhance the effectiveness and safety of new treatments over current options, researchers must focus on two key areas. First, they need to refine PEG-conjugated thrombolytic agents to boost their activity and extend their half-life, thereby improving the relevance and translatability of preclinical models. Second, it is crucial to advance pharmacological approaches by identifying specific targets for these agents, optimizing their pharmacokinetic properties, and assessing their thrombotic action in multiple mouse models of venous thrombosis. This comprehensive approach will better capture the complexity of venous thrombosis and strengthen the foundation for translational research, ultimately leading to more effective and tolerable therapies for patients.

#### 6.3.3. Liposomes

Liposomes are spherical vesicles with a size range of 50 to 500 nm in diameter, and are primarily made of phospholipids such as soybean phosphatidylcholine, synthetic dialkyl, or trialkyl lipids [176]. During liposome preparation, cholesterol is incorporated into liposomes not only to modulate membrane permeability and fluidity [177], but also to improve the stability of the vesicle membrane in the presence of biological fluids such as blood and plasma. Liposomes containing polymers [178] and even membrane proteins [179] offer multiple benefits to overcome the limitations of both the traditional drug dosage forms and conventional liposomes, the first generation of liposomes. These benefits include prolonged circulation half-life, enhanced localization of drug in the desired tissue, enhanced localization of drug in the desired tissue [180], and improved effectiveness of the encapsulated drug. Moreover, the incorporation of phospholipids-attached PEG [181] into the chemical structure of liposomes represents another approach to enhance the pharmacokinetic properties and biodistribution profiles of liposomes. Small molecules make up about 90% of all prescriptions [182]. The therapeutic effect of small hydrophilic molecules is limited by their poor pharmacokinetics and biodistribution. A recent review delves into obstacles in hydrophilic drug delivery and elucidates the design of liposomal carriers for small hydrophilic molecules that can provide solutions [183]. Hydrophobic and hydrophilic characteristics of liposomes create two distinct compartments in which drug molecules are encapsulated in the aqueous compartment of the vesicles or in the lipophilic membrane compartment. Liposomes are proven to be ideal carriers for a range of drugs as well as diagnostic and targeted drug delivery applications. The FDA has approved a wide range of liposomal-based drug delivery systems for treating and preventing human diseases [184].

Liposomes loaded with various antithrombotic drugs are considered to lack proven clinical applications [185]. Currently, a number of liposome formulations are in preclinical use to deliver thrombolytic drug molecules for treating arterial thromboembolic diseases, myocardial infarction, and stroke, as well as VTE, deep vein thrombosis, and pulmonary embolism (Table 4). Staphylokinase (SAK), an indirect activator of plasminogen, is a secretory enzyme produced by staphylokinase [186]. An in-depth analysis of the impact of the interaction of the SK variants with antimicrobial peptides has been explored [187]. However, the involvement of staphylokinase variants in activating plasminogen remains a dilemma. Encapsulated staphylokinase in temperature-sensitive liposomes appears to be promising [188]. Low-molecular-weight heparin (LMWH) in an intermediate dose outperforms other anticoagulant drugs for the prevention of VTE [189]. However, it requires systemic routes of administration (e.g., injection). The encapsulated LMWH-liposome construct exhibits a long half-life in circulation [190]. Cationic flexible liposomes demonstrate a higher entrapment efficiency of LMWH compared to neutral and anionic flexible liposome formulations [191]. Topical application of LMWH-cationic flexible liposomes deeply penetrated into the skin [191], thereby suggesting this formulation may be a compelling ally for the treatment of superficial thrombosis. Over a decade later, inhalable distearoyl-sn-glycero-3-phosphoethanolamine (DSPE) and long-circulating pegylated liposomes of LMWH are found to be effective in reducing thrombus weight, and are suggested to be a potential noninvasive approach for DVT and PE treatment [137]. However, there is no clinical evidence yet to corroborate that encapsulated HMWH-liposomes are effective therapeutics in humans.

#### 6.3.4. Echogenic Liposome

Several targetable, drug-carrying echogenic liposome preparations have been developed following reports of multiple patient deaths due to the injection of ultrasound contrast agents [192,193]. Echogenic liposomes (ELIPs) are lipid-bilayer agents [194] that contain gas-filled monolayered microbubbles and sonosensitive vesicles. Gas vesicles are capable of scattering sound waves and, therefore, produce ultrasound contrast [195]. ELIPs are not only an ultrasound contrast agent, but also an ultrasound-activating drug delivery system [196,197]. While ELIPs were initially loaded with methotrexate (MTX), they have been loaded with a variety of therapeutic agents, such as hydrophilic and lipophilic drugs, proteins, and peptides. To learn more about echogenic liposomes, the latest progress in echogenic liposome preparations, characteristics, and applications can be found in an excellent review of drug delivery [198]. Single chain urokinase-tPA (sutPA)-loaded ELIPs specifically bind to fibrin (Table 4) [199]. Ultrasound significantly increased lytic treatment efficacy for recombinant tPA-loaded ELIPs [151]. tPA-loaded ELIPs are suitable for ultrasound-detectable local drug delivery [200]. tPA-loaded ELIPs have the potential to serve as a therapeutic antithrombotic agent in the treatment of DVT or PE [201]. Moreover, it allows a lower dosage of thrombolytic drugs to be used. Since the effectiveness of tPA-loaded ELIPs has been tested, exploring the effect of drug interactions and improving the understanding of risk factors associated with the pathogenic changes that occur in the blood vessel wall is important in developing more effective antithrombotic drugs.

#### 6.3.5. Polymeric Nanoparticles

Polymeric nanoparticles can be used to deliver thrombolytic drugs. Several factors, including size, surface charge, and the presence or absence of a polymer coating, influence the clearance and biodistribution of nano- and microcarriers [202]. Lipid-based nanocarriers are liposomes, which were discussed earlier. Synthetic and natural polymers are employed in the design of nano- and micro-carriers for thrombolytic drugs. These polymers offer the advantage of greater resistance to mechanical stress compared to lipid-based carriers [203].

Inorganic nanocarriers used for the delivery of thrombolytic drugs are primarily represented by magnetic nanoparticles. Therapeutic agent-loaded magnetic nanoparticles are drawn to the thrombus surface in response to an external magnetic field. Magnetic nanoparticles are biodegradable and participate in the iron homeostasis of the body [204,205,206,207]. Fithri et al. [149] worked on developing gold-iron oxide nanoparticles (AuIONP(+)) for thrombosis. AuIONP(+), a theranostic agent (referred to as a combination of diagnostic and therapeutic agents), in antithrombotic therapy due to the site-specific delivery of antithrombin agents to the targeted activated platelets, showed excellent antithrombotic activity. While AuIONP(+) is capable of binding to thrombi, including the exhibition of excellent properties for photoacoustic imaging of thrombi, good biocompatibility, as well as the signs of successful thrombolysis and complete thrombus elimination, was not achieved [149]. However, AuIONP(+) partially restores blood flow. Zhang et al. [208] developed a novel multifunctional nanoplatform for thrombolytic therapy. This mesoporous nanoplatform has the following two key features: (1) near-infrared, alternating magnetic field and (2) urokinase plasminogen activators (uPA)-loaded metal−organic-framework (MOF)-derived carbon-based materials (referred to uPA@CFs). uPA@CFs exhibit near-infrared (NIR)-stimulated uPA release and heat the thrombosis at DPV via alternating magnetic field. uPA@CFs showed a high loading ability of the uPA, good NIR-stimulated uPA release, and good photothermal conversion efficiency, addressing the issue of low drug utilization rates [208].

Zhong et al. [209] worked on ultrasound-responsive phase-transitional nanoparticles for thrombolysis using Fe3O4-PLGA-PFH-CREKA nanoparticles (NPs). This well-developed phase transition agent responded effectively to low-intensity focused ultrasound (LIFU) by triggering the vaporization of liquid perfluorohexane (PFH) to achieve thrombolysis and CREKA peptide, which binds to fibrin in the thrombus, enabling targeted imaging and effective thrombolysis. LIFU-responsive photothermal (PT) thrombolysis reduced thrombus burden post-irradiation without impairing vascular tissue [209].

#### 6.3.6. Dendrimers

Delivering accurate doses can be challenging. Precise control over the distribution of a drug in the body is a strategy to promote the accurate dose at a specific tissue site while minimizing deleterious side effects. Dendrimers, as vehicles for drug delivery systems, provide high-capacity loading and sustained release profiles. Dendrimers are highly valuable because they can address the challenges of clearance and inactivation; thereby, dendrimer-based drug delivery has attracted increasing interest for numerous reasons. Due to their site-specific delivery capability, various dendrimers and dendrimer derivatives [210] have been developed [211] and utilized for a variety of diseases [212] since their discovery in 1978 [213]. Unlike traditional polymers, dendrimers are synthetic macromolecules of nanometer dimensions. Dendrimers have attractive properties, including high solubility, low viscosity, great mixability, biodegradability, good biocompatibility, as well as high reactivity. Dendrimers have been attracting a growing interest mainly because of their characteristics, including a regularly branched tree-like structure [214], well-defined globular structures [215], the ability to attach functional groups to the chain ends of dendrimers, variable chemical composition, capability of storing [216] various carbon-rich guests with hydrophilic or hydrophobic nature via electrostatic interaction or covalent bonding, and high biological compatibility. Dendrimers are customizable nanotechnologies. The delivery of the therapeutic agents at specific sites in the body has been attained by surface modification of the terminal ends of dendrimers using numerous targeting moieties such as folic acid [217], peptides [218], monoclonal antibodies [219], and sugar groups [220]. These features allow them to be an ideal carrier for drug delivery and hold promise in increasing selectivity, reducing off-target effects, and decreasing unwanted toxicities.

Evidence indicates that amine-terminated dendrimers cause disseminated intravascular coagulation-like conditions via acting on fibrinogen in a thrombin-independent manner [158] and cationic dendrimers have been shown to activate platelets, leading to disrupted morphology [221]. Interestingly, cationic dendrimers are capable of inhibiting the extrinsic activation pathway of the coagulation system, leading to prolonged prothrombin time and suppressing thrombin generation in plasma [222]. Neutral dendrimers [223] are not hemolytic and do not alter platelet morphology or their function in vitro. Dendrimers with low molecular weight [224] improve the storage time of platelets. Dendrimers have emerged as vectors for delivering clot-busting therapeutics to the target site, concurrently preventing access to the nontarget site due to the availability of multiple functional groups. Tissue plasminogen activator, a clot-busting drug, is used in treating stroke. The tPA-dendrimer complex is found to have the highest clot-dissolving activity compared to that of control tPA (Table 4) [154]. Nattokinase is used for thrombolytic therapy. It has tPA activity and is capable of directly digesting fibrin via limited proteolysis. Evidence indicates that the nattokinase–dendrimer complex possesses a great thrombolysis ability both in vitro and in vivo (Table 4) [225]. Future studies are warranted to address whether the thrombolytic-neutral dendrimer complex is more effective than those of the current anticoagulants and better understand the complex pharmacology and interaction of the thrombolytic-neutral dendrimer complex within preclinical models (small and large animals) and their respective biological systems, including thrombus microenvironment.

#### 6.3.7. Mechanically Activated Nanotherapeutics

Restrictions of normal blood flow are due to stenosis, thrombosis, embolism, or hemorrhage, which can lead to poor circulation. The leading causes of circulation problems are peripheral vascular disease, atherosclerosis, stroke, placental microthrombosis, and VTE. During the past decade, various types of nanoparticles have been prepared to accurately identify the thrombus and to be used for targeting various analytes such as antiplatelet and thrombolytic drugs. Herein, we highlight recent developments on stimuli-responsive nanoparticles (e.g., mechanosensitive nanoparticles), nanogels (e.g., hydrogels), and multicomponent nanoparticles (e.g., fucoidan (Fuc, a P-selectin ligand)-based core-shell nanoparticles). Our goals are: (1) to highlight the successful development of novel drug delivery systems that not only allow the drug to be localized on the thrombus to cause thrombolysis but also are characterized to reduce drug toxicity and adverse effects, and (2) to underscore how certain nanoparticles loaded with antithrombotics alone or in combination with anti-inflammatory, antioxidant, and endothelial protective agents could potentially become more effective treatments for VTE and lessen the harms associated with side effects of anticoagulants and antithrombotics. Below, we focus on the update of these nanoparticles that possess high potential for the treatment of VTE. Structures, properties, and recent applications of hydrogels have been addressed before in excellent work by others [226,227].

Mechanosensitive micrometer-sized aggregates of nanoparticles function as stimuli-responsive drug carriers, designed to release their payload within blood vessels by high-fluid shear stresses at sites of vascular obstruction [161,162]. The intravenous administration of shear-activated micrometer aggregates of nanoparticles coated with tPA induces rapid clot dissolution in a mesenteric injury model [161]. It is well-established that the blood shear stress at the thrombus site is significantly greater than the force that the blood flow exerts on the vessel wall of normal arteries. A positive correlation between platelet deposition at the thrombus site and the increasing shear rate is reported [228,229,230], highlighting that the activation and aggregation of platelets are due to high shear stresses. However, characteristics of the underlying vessel, such as the stenotic length and contraction angle of stenosis, appear to cause shear-dependent platelet aggregation formation [231]. Thus, thrombus formation and its complications are complex, multifactorial conditions in which both non-genetic and genetic factors are involved. Zhang et al. [232] explored the development of a novel drug delivery platform using the antiplatelet drug tirofiban (TI) and the thrombolytic drug urokinase (UK) in fucoidan (Fuc, a P-selectin ligand)-based core-shell nanoparticles (referred to UK@Fuc-TI/PPCD). P-selectin is expressed on both activated endothelial cells and activated platelets [233] under inflammation-related pathologic states [234], during thrombus development [235], and cancer progression [235]. The key features of the UK@Fuc-TI/PPCD platform are its ability to bind specifically to its target antigen, P-selectin, and its mechanosensitive capability that preferentially releases its payload under elevated shear stress. Despite its limited characterization, the use of UK@Fuc-TI/PPCD in preclinical studies as a therapeutic to dissolve blood clots looks promising. The emerging role of inflammation and ROS in metabolic disorders [236] and the interplay between inflammation and thrombosis [237] has generated increasing interest in developing an advanced drug delivery system that is activated at sites of thrombus development to circumvent these problems. The focus here is on specific areas in research concerning complications of thrombosis (inflammation, reactive oxygen species (ROS) production, and endothelial perturbation) using hydrogels for therapeutic delivery. Hydrogel, the first biomaterial, was initially used to make the first soft contact lens [238]. While ordinary hydrogels exhibit swelling–deswelling behavior in water, advanced hydrogels with tunable properties [239,240,241,242] are designed to change their properties in response to a subtle change in a tissue microenvironment. Hydrogel-based drug delivery systems are clinically offering targeted delivery and tissue repair at the site of injury, such as wound dressing [243], plastic surgery, and as tissue sealant [244]. Injectable hydrogel appears to be a promising candidate for wound healing therapy. Evidence indicates that ibuprofen-loaded heparin-modified thermosensitive hydrogel may be capable of inhibiting inflammation and improving wound healing [245]. Inflammation-responsive drug-loaded hydrogels in combination with encapsulation with vancomycin (an antibiotic)-conjugated silver nanoclusters and pH-sensitive micelles loaded with nimesulide, a nonsteroidal anti-inflammatory drug, exhibit wound healing in streptozotocin-induced diabetes in rats through antibacterial, anti-inflammatory processes [246]. Similarly, injectable hyaluronic acid hydrogel in combination with antioxidant gallic acid-grafted hyaluronic acid promotes the microglia polarization to M2 phenotype and exhibits antioxidant activity [247]. To learn more about nanotherapeutics, the reader is referred to several excellent reviews of nanoparticles and their associated challenges for therapeutic delivery [124,226,248,249,250,251]. The recent advances achieved with thrombus-targeting, inflammation-targeting, antioxidant-targeting, and endothelial-targeting nanocarriers could lay the foundation to accelerate the development of polymeric nanocarriers containing a combination of antithrombotic, anti-inflammatory, antioxidant agents for the treatment of thrombosis and the clinical translation of each of these polymeric nanocarriers.

#### 6.3.8. Platelet-Based Drug Delivery System

Platelets are the smallest anucleated cells that circulate within the blood circulatory system. They respond to both intravascular and blood vessel environmental signals. They are terminally differentiated and may have a circulatory life span of up to seven days. Platelets, or thrombocytes, serve as sentinel cells to detect vascular injury in many living species throughout the evolutionary tree. Central to their function is their ability to communicate continually with normal blood vessel endothelial cells via paracrine signaling. With excessively low levels of the paracrine signaling due to endothelial dysfunction [252] or an injury, quiescent platelets that circulate in the blood stick to the damaged tissue and they transform shape, known as platelet activation. The activated platelets recruit and activate more platelets to the site of vascular injury through a combination of paracrine- and autocrine-mediated signaling mechanisms via the release of platelet activators, namely thromboxane A2, adenosine diphosphate (ADP), and calcium from platelets. The activated platelets also establish a feed-forward mechanism that amplifies the coagulation cascade, allowing for rapid generation of thrombin. Formed thrombin causes platelets to release the contents of their granules and induce platelet plug formation, among many other functions as mentioned earlier. The platelet plug, also known as aggregation, is important for a wide range of protective physiological responses, including coagulation, complementation, inflammation, and immune, which are effective against a diverse variety of threats. Platelet aggregation is essential for healing to occur because they adhere to one another to form a temporary hemostatic plug, a thrombus, to seal an injury, initiating the process of hemostasis. Over the past three decades, as we have learned more about how the abovementioned physiological processes are pivotal for the progression of wound healing and tissue repair, it has become clear that there is an extensive crosstalk among them. Activation of one system influences the functional ability and activity of the other. For instance, excess platelet activation is linked to the thrombo-inflammatory process [253,254]. Elevated C3, a complement component, levels are implicated as a DVT risk factor in humans [255]. Unregulated immune and inflammatory processes can promote thrombosis [256]. Besides the coagulation system, abnormal activation of complementation, immune, and inflammatory systems are key players in the pathophysiology of DVT. A detailed summary of platelet adhesion, activation, and aggregation is reviewed elsewhere [257].

Not only do platelets control thrombosis but also mediate VTE, stroke, and myocardial infarction [258]. The innate roles of platelets in DVT are supported by the fact that aspirin (Table 1), an inhibitor of thromboxane A2 (a potent activator of platelets) synthesis, reduces VTE in patients undergoing orthopedic surgery [259]. Adhesion of platelets to the site of injury is mediated via the interaction between GPIbα, a platelet membrane receptor, and the von Willebrand factor (vWF) exposed on the endothelial surface [260], suggesting vWF mediates platelet activation. Evidence also shows that vWF mediates platelet aggregation through binding to GPIIb/GPIIIa, a platelet membrane glycoprotein [261,262], as described below. Evidence for a consistent role of platelet–endothelial interaction is described in the venous thrombus [256]. Elevated generation of ROS promotes platelet recruitment to the developing venous thrombus, leading to thrombus growth [263]. Regardless of triggers, activation of platelets results in conformational changes (inside-out signaling) of GPIIb/IIIa, a platelet membrane protein. This conformational altered GPII/IIIa has a specific binding affinity for soluble plasma fibrinogen or other plasma molecules (vWF, fibronectin, and vitronectin) [264] with the RGD domain. The bivalent fibrinogen serves to connect two adjacent activated platelets through its specific binding site on platelets [265], allowing platelet aggregation and thrombus formation. GPIIb/IIIa has a pivotal role in the formation of platelet thrombi. The inhibitors of GPIIb/IIIa inhibit shear-induced platelet aggregation (Table 1) [266].

In view of the above studies conducted on platelet properties, significant progress in the research on biomimetic drug delivery systems has been achieved. The platelet (P) membrane is attached onto the surface of a nano-drug delivery system, poly(lactic-co-glycolic acid; PLGA), an FDA-approved polymeric core [267], to generate P-camouflaged polymeric nanoparticles (nanoplatelets), PNP [164]. Then, a site-specific conjugation technique is used that involves the conjugation of recombinant tPA (rtPA) to the activated sulfhydryl groups residing on the external surface of the platelet membrane of nanoplatelet to construct PNP-PA (Table 4). This construct aims to help target the delivery of thrombolytic drugs to local thrombus sites. PNP-PA is found to have a dual benefit of possessing the major membrane adhesion-associated proteins (GPIIb/IIIa and P-selectin), thereby improving PNP-PA recruitment to the thrombus site, and the ability to achieve targeted thrombolysis. PNP-PA exhibits a low risk of bleeding complication in different animal models of thrombosis [164]. Although anticoagulants are generally used to treat DVT or PE, PNP-PA appears to be a promising agent to treat both arterial and venous thrombi.

Using a conceptually derived strategy in a platelet-based drug delivery system, PNP-loaded lumbrokinase (LBK), a fibrinolytic enzyme, is constructed to form nanoplatelets (PNPs/LBK) to target the thrombus site [268]. Like PNP-PA, after binding of PNPs/LBK to the active platelet integrin GPIIb/IIIa and P-selectin on thrombus, LBK is released. LBK is released by phospholipase-A2-induced vesicle destabilization. Administration of PNPs/LBK has little effect on the bleeding time compared to free LBK. PNP/LBK is a promising antifibrinolytic agent, which can effectively target the thrombus site and prevent hemorrhagic episodes.

## 7. Future Implications and Research Opportunities

The development of antithrombotic drugs and advanced delivery systems presents significant opportunities to improve the management of VTE. VTE is strongly related to age, with the highest incidence being seen among the elderly. As chronic diseases among older adults increase, the risk of VTE and its associated complications becomes more pronounced, necessitating more effective and safer treatment options [269]. Future research should focus on identifying and validating novel targets within the coagulation cascade, particularly those that can provide a more precise modulation of thrombin activity in elderly patients with multiple comorbidities. Next-generation anticoagulants with enhanced efficacy and safety profiles are crucial, especially for this vulnerable population. Understanding the molecular mechanisms underlying individual variability in response to antithrombotic therapy could lead to more personalized treatment approaches, optimizing therapeutic outcomes for diverse patient populations across different age groups and comorbidity profiles [270]. This personalized approach is particularly relevant for the aging population, where tailored interventions can significantly impact quality of life and reduce the burden on healthcare systems [271]. The integration of advanced drug delivery systems, such as nanoparticle-based carriers and targeted delivery mechanisms, holds promise for enhancing the specificity and bioavailability of antithrombotic agents [272]. These technologies could enable localized drug delivery, reducing systemic exposure and associated side effects, which is especially beneficial for older patients who may be more susceptible to adverse drug reactions. As discussed in previous sections, of particular interest is the development of nanocarriers in thrombolytic drug delivery, which have shown significant potential in preclinical studies for the treatment of VTE. Collaborative efforts between academic institutions, industry, and regulatory bodies will be vital to accelerate the translation of these innovations from bench to bedside, ultimately advancing the standard of care for patients with VTE.

## Figures and Tables

**Table 1:** Selected antithrombotic drugs.

Drug	Elimination	Risk	Comments
**Anticoagulants**			
**Direct Xa inhibitors** ○ Apixaban ○ Betrixaban ○ Edoxaban ○ Rivaroxaban	○ Fecal, 25% kidneys ○ Hepatobiliary system ○ Primarily via kidneys ○ 2/3 liver, 1/3 kidneys	○ Major bleeding ○ Discontinued ○ Bleeding ○ Bleeding	Approved • Prevention of stroke • Prevention of systemic embolism in nonvalvular atrial fibrillation • Prevention of DVT and PE • Prevention of recurrent DVT and PE following initial treatment
**Direct thrombin inhibitors** ○ Argatroban ○ Bivalirudin ○ Dabigatran ○ Desirudin ○ Hirudin	○ Primarily fecal, kidney ○ Proteolytic cleavage, 20% kidneys ○ Fecal, 85% kidneys ○ Primarily via kidneys ○ Primarily via kidneys	○ Bleeding ○ Bleeding ○ Bleeding ○ Bleeding ○ Severe bleeding	Approved • Stroke prevention in nonvalvular atrial fibrillation • VTE prophylaxis
**Indirect thrombin inhibitors** ○ Dalteparin ○ Enoxaparin ○ Fondaparinux ○ Heparin-unfractionated	○ Liver, GI, 20% kidneys ○ Primarily via kidneys ○ Primarily via kidneys ○ Primarily via reticuloendothelial system		Approved • To treat and prevent thrombotic disorders (DVT, atrial fibrillation, PE) • Acute coronary syndrome • Heparin-induced thrombocytopenia (HIT) • DVT and PE
**Vitamin K antagonists** ○ Warfarin	○ 92% via kidneys		Approved • For the prevention and treatment of venous and arterial thrombosis
**Antiplatelets**			
**COX inhibitors** ○ Aspirin ○ Other NSAIDs	○ Mainly via kidneys		Approved • Thromboembolic events • Acute coronary syndrome • Acute ischemic stroke • Post percutaneous coronary intervention (PCI) • Reduce the risk of heart attack
**GPIIb/IIIa inhibitors** ○ Abcixvimab ○ Eptifibatide ○ Tirofiban	○ Mainly via kidneys ○ 50% via kidneys ○ 65% kidneys, 25% fecal	○ Bleeding	
**P2Y12 inhibitors** ○ Cangrelor ○ Clopidogrel ○ Prasugrel ○ Ticagrelor ○ Ticlopridine	○ Primarily via kidneys ○ 50% kidneys, 45% fecal ○ 70% kidneys, 25% fecal ○ Primarily via liver ○ 60% kidneys, 23% fecal		
**Phosphodiesterase inhibitors** ○ Cilostazol ○ Dipyridamole	○ Primarily by metabolism, 95% liver		
**Thrombolytics** **Plasminogen activators** ○ Retallies ○ Streptokinase ○ Tissue plasminogen activator	○ Primarily by liver ○ Primarily by liver ○ Primarily by liver		Approved • The emergency treatment of stroke and heart attack • Acute PE • Ischemic stroke • Myocardial infarction

**Table 2:** Compelling indications for antithrombotic drugs are based on benefits from outcome studies or clinical guidelines.

	Recommended Antithrombotic Drugs			
Compelling Indications	Anticoagulants	Antiplatelets	Thrombolytics	Ref.
**Coronary artery disease (CAD)**	Anticoagulans	Clopidogrel	Thrombolytics	[74,75,76]
**CAD undergoing percutaneous coronary intervention**		Prasugrel, ticagrelor		[77]
**Chronic kidney disease** ○ **Mild** ○ **Moderate** ○ **Severe**	○ Warfarin and direct oral anticoagulants ○ Warfarin and direct oral anticoagulants ○ Direct oral anticoagulants (rivaroxaban)			[78,79] [80] [80] [81,82]
**Diabetes**	Non-vitamin K antagonist oral anticoagulants	Purinergic receptor antagonists	Thrombolytic	[83,84,85,86]
**Knee replacement,** **orthopedic surgery**	Warfarin, Rivaroxaban, Apixaban, LMWH, Fondaparinux	Aspirin	Urokinase thrombolysis	[87,88,89,90]
**Heart failure** **Spontaneous coronary artery dissection (SCAD)**	Warfarin, oral anticoagulants	Aspirin and clopidogrel		[91,92] [93]
**Liver disease**	Alteplase			[94]
**Recurrent VTE** ○ **DVT** ○ **PE**	Heparins, apixaban, fondaparinux			[95,96]

**Table 4:** Characteristics of the nano-drugs and the in vitro thrombotic and fibrinolytic activity of these nano-drugs compared to their respective non-conjugated fibrinolytic activator alone.

Nano-Drugs	Characteristics	Outcome	Ref.
**PEGylation** 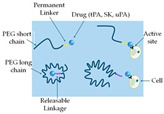	PEG-tPA PEG-UK PEG-SK PEG-SAK PEG-maleimide-(poly-SAK)	Reduced proteolytic activity Slower inhibition kinetics by PAI-1 Increased fibrinolysis Resistant to plasmin cleavage Increased fibrinolysis Slightly increased fibrinolysis Increased bioactivity	[131] [132] [133,134] [135] [136]
**Liposome** 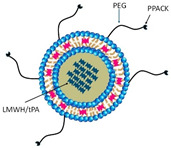	A circular-shaped diacyl-chain phospholipids/phospholipid-attached PEG with cholesterol liposomal LMWH liposomal t-PA PPACK-liposome	Reduce thrombus weight Improve thrombolytic efficacy, reduce tPA-induced hemorrhage Prolong inhibition of thrombosis, reduced systemic side effects	[137] [138] [139,140]
**Echogenic liposome and polymeric nanoparticles** 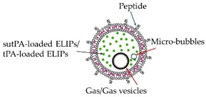	tPA-loaded ELIPs NIR-stimulated uPA release Magnetic nanoparticles Ultrasound-guided RDG-modified ELIPs	Enhance thrombolytic efficiency Significant thrombolysis Prolong circulating tPA Improve thrombolytic efficacy Minimize off-target effects Similar thrombolysis, reduce the dose of tPA Complete thrombus elimination Effective thrombolysis in a rat embolism model Enhanced thrombolytic efficacy of tPA Improve recanalization rate	[141,142,143] [144] [145] [141,146,147] [148] [148] [149] [150] [151] [152]
**Dendrimer** 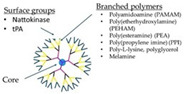	tPA-dendrimer complex Nattokinase–dendrimer complex LMWH–dendrimer complex Poly(amidoamine) dendrimers Poly(lysine) dendrimers	High clot-dissolving activity Effective thrombolytic effect Prevents DVT Induce fibrinogen aggregation, contribute to the in vivo DIC, produce rapid coagulation Ideal carriers of protein drugs	[153] [154] [155,156] [157] [158] [156]
**Mechanically activated nanotherapeutics** 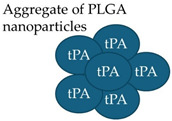	tPA-PLGA shear-activated nanoparticle (tPA-SA-NP) complex tPA-loaded SA-NP and temporary endovascular bypass (TEB)	Rapid clot dissolution Increase recanalization, reduce distal embolization	[159,160] [161,162] [163]
**Platelet-based drug delivery system** 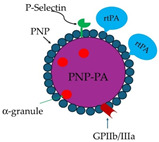	rtPA-PNP-PA	Thrombolysis	[164]

## Data Availability

The original contributions in the study are included in the article, further inquiries can be directed to the corresponding author.

## Abbreviations

ACCP	American College of Chest Physicians
ADP	Adenosine Diphosphate
AIDS	Acquired Immunodeficiency Syndrome
APTT	Activated Partial Prothrombin Time
ASOIs	Antisense Oligonucleotide Inhibitors
AuIONP+	Gold-Iron Oxide Nanoparticles
C1-Inhibitor	C1-Esterase Inhibitor
CAD	Chronic Artery Disease
CKD	Chronic Kidney Disease
COX	Cyclooxygenase
CREKA	Cys-Arg-Glu-Lys-Ala
CRP	C-Reactive Protein
DIC	Disseminated Intravascular Coagulation
DOACs	Direct Oral Anticoagulants
DSPE	1,2-Distearoyl-Sn-Glycero-3-Phosphoethanolamine
DVT	Deep Vein Thrombosis
ECM	Extracellular Matrix
ELIP	Echogenic Liposomes
FDA	U.S. Food and Drug Administration
FDP	Fibrin Degradation Products
Fibrinogen	Factor I
FIX	Factor IX
FIXa	Activated Factor IX
Fuc	Fucoidan
FVII	Factor VII
FVIIa	Activated Factor VII
FVIII	Factor VIII
FVIIIa	Activated Factor VIII
FX	Factor X
FXa	Activated Factor X
FXI	Factor XI
FXIa	Activated Factor XI
FXII	Factor XII
FXII	Factor XIII
FXIIa Hageman Factor	Activated Factor XII
FXIIIa	Activated Factor XIII
GI	Gastrointestinal
GP IIb/IIIa	Glycoprotein IIb/IIIa
HMWH	High-Molecular-Weight Heparin
INR	Target International Normalized Ratio
LBK	Lumbrokinase
LIFU	Low-Intensity Focused Ultrasound
LMWH	Low-Molecular-Weight Heparin
MOF	Metal-Organic-Framework
MTX	Methotrexate
NHLBI	National Heart, Lung, and Blood Institute
NIR	Near-Infrared
NPs	Nanoparticles
NSAIDs	Non-Steroidal Anti-Inflammatory Drugs
P2Y12	Purinergic Receptor Type Y, Subtype 12
PA	Plasminogen Activator
PAI	Plasminogen Activator Inhibitor
PE	Pulmonary Embolism
PEG	Polyethylene Glycol
PFH	Perfluorohexane
PLGA	Poly(Lactic-Co-Glycolic Acid)
PNP	P-camouflaged Polymeric Nanoparticles
polyP	Platelet-Derived Polyphosphate
PPACK	D-phenylalanyl-L-prolyl-L-arginyl-chloromethyl Ketone
PPCD	Doxorubicin-Polymer Conjugates
Prothrombin	Factor II
PSGL-1	P-selectin Glycoprotein Ligand-1
PT	Prothrombin Time
RGD	(Arg-Gly-Asp) Peptide
ROS	Reactive Oxygen Species
rtPA	Recombinant tPA
SAK	Staphylokinase
SCAD	Spontaneous Coronary Artery Dissection
SK	Streptokinase
SNP	Single Nucleotide Polymorphism
TAFI	Thrombin-Activated Fibrinolysis Inhibitor
TF	Tissue Factor
TFPI	Tissue Factor Pathway Inhibitors
Thrombin	Activated Factor II
tPA	Tissue-Type Plasminogen Activator
TT	Thrombin Time
U.S.	United States
UK	Urokinase
UK@Fuc-TI/PPCD	Urokinase (UK) in Fucoidan-Based Core-Shell Nanoparticles
uPA	Urokinase-Type Plasminogen Activator
uPA@CFs	Urokinase Plasminogen Activators (uPA)-Loaded Metal-Organic-Framework (MOF) Derived Carbon-Based Materials
VTE	Venous Thromboembolism
vWF	Von Willebrand factor
